# The roles of mesenchymal stem cell-derived exosomes in diabetes mellitus and its related complications

**DOI:** 10.3389/fendo.2022.1027686

**Published:** 2022-10-21

**Authors:** Mengmeng Yang, Jun Chen, Li Chen

**Affiliations:** ^1^ Department of Endocrinology, Qilu Hospital, Shandong University, Jinan, China; ^2^ Institute of Endocrine and Metabolic Diseases of Shandong University, Jinan, China; ^3^ Key Laboratory of Endocrine and Metabolic Diseases, Shandong Province Medicine & Health, Jinan, China; ^4^ Jinan Clinical Research Center for Endocrine and Metabolic Diseases, Jinan, China

**Keywords:** diabetes mellitus, complications, mesenchymal stem cells, exosomes, β-cell

## Abstract

Diabetes mellitus is a type of metabolic disease characterized by hyperglycemia, primarily caused by defects in insulin secretion, insulin action, or both. Long-term chronic hyperglycemia can lead to diabetes-related complications, causing damage, dysfunction, and failure of different organs. However, traditional insulin and oral drug therapy can only treat the symptoms but not delay the progressive failure of pancreatic beta cells or prevent the emergence of diabetic complications. Mesenchymal stem cells have received extensive attention due to their strong immunoregulatory functions and regeneration effects. Mesenchymal stem cell-derived exosomes (MSC-Exos) have been proposed as a novel treatment for diabetic patients as they have demonstrated superior efficiency to mesenchymal stem cells. This review summarizes the therapeutic effects, mechanisms, challenges, and future prospects of MSC-Exos in treating diabetes mellitus and its related complications. This review supports the potential use of MSC-Exos in future regenerative medicine to overcome the current difficulties in clinical treatment, particularly in treating diabetes.

## 1 Introduction

Diabetes mellitus is (DM) a metabolic disease characterized by hyperglycemia. It is primarily divided into Type 1 diabetes mellitus (T1DM), which occurs due to absolute deficiency in insulin secretion caused by autoimmune destruction of pancreatic beta cells (β-cells). Additionally, Type 2 diabetes mellitus (T2DM) is caused by insulin resistance or islets β-cells dysfunction (1, 2). Diabetes has become the ninth leading cause of mortality, with the total number of people with diabetes quadrupled in the past three decades (3). More than 440 million people are currently affected by the diabetes epidemic, with approximately 1 in 10 adults having diabetes, with T2DM accounting for 90% (3). Long-term chronic hyperglycemia can lead to diabetes-related complications, causing damage, dysfunction, and failure of multiple organs and tissues, such as the eyes, kidneys, nerves, heart, blood vessels, etc. Most patients with T2DM have at least one of these complications, with cardiovascular complications being the most common cause of mortality (3). Meanwhile, T1DM is closely associated with various microvascular and macrovascular complications (4). The rising prevalence and serious complications have made diabetes a huge challenge that significantly jeopardizes public health.

Insulin injection and oral hypoglycemic medications are the traditional treatments for diabetes. However, these treatments cannot slow down the progressive failure of pancreatic β-cells and prevent the emergence of diabetic complications. Recently, researchers have focused on mesenchymal stem cells (MSCs) therapy as a potential treatment option for diabetic patients. MSCs are the heterogeneous subset of stromal stem cells that can be isolated from various tissues, including the umbilical cord, amniotic fluid, menstrual blood, bone marrow, adipose tissue, and others. MSCs exhibit self-renewal and the potential for multi-lineage differentiation, including mesodermal lineages, such as adipocytes, osteocytes, chondrocytes, etc. (5, 6). MSCs have emerged as the most promising source of cells for transplantation in recent years due to their immunomodulatory (7), paracrine (8), and trans-differentiation regulatory functions (9). Emerging studies have reported that MSCs are crucial in treating diabetes and its related complications (10–12). However, there are limitations in MSCs cell therapy, namely organ residence (13), limited utilization (14), thrombogenesis (15), low survival rate *in vivo* (16), and tumorigenicity potential (17, 18). Accumulating evidence indicates that MSCs exert their therapeutic effects mainly through the paracrine mechanisms, prompting extensive studies on mesenchymal stem cell-conditioned medium (8, 19, 20). Mesenchymal stem cell-derived exosomes (MSC-Exos) have been proven to be equally effective as MSCs in treating diabetes and related complications (21–23). In some studies, MSC-Exos have demonstrated a superior therapeutic and regenerative effect in treating T1DM compared with the parental cells (24). Furthermore, it is easier to maintain exosomes than MSCs, in addition to being a safer option due to the fewer membrane-bound proteins and lack of direct tumorigenicity (25). The ability of cell-free exosome therapy to circumvent the aforementioned shortcomings of MSCs transplantation combined with its excellent therapeutic effects and safety makes it a new strategy for the treatment of diabetes and diabetic complications. This review summarizes the biological characteristics of exosomes and their applications in the treatment of diabetes and its related complications. This review also elucidates the potential underlying mechanisms and we discuss the challenges related to their applications.

## 2 Properties of MSCs

MSCs are a class of pluripotent stem cells belonging to the mesoderm, which have all the common characteristics of stem cells, namely self-renewal and multidirectional differentiation ability. MSCs can differentiate into osteogenic, chondrogenic, and adipogenic lineages. Studies have proven that MSCs play important roles in many diseases including diabetes and related complications.

The most prevalent types of MSCs are mainly derived from bone marrow, adipose tissue, and perinatal tissues (human umbilical cord, umbilical blood, amniotic membrane, placenta, etc.). The biological properties of MSCs from various tissue origins vary. Both bone marrow MSCs (BMMSCs) and adipose MSCs (AMSCs) have trilineage differentiation potential. However, BMMSCs exhibit enhanced osteogenic and chondrogenic differentiation, whereas AMSCs are typically more likely to exhibit an adipogenic differentiation (26). Interestingly, umbilical blood MSCs (UCBMSCs) and placenta MSCs can differentiate into only two lineages (27). Even though there are conflicting results regarding the differentiation potential of perinatal tissues MSCs (PTMSCs), Marianna et al. confirmed that UCBMSCs have a lower adipogenic potential (28). In addition to the differentiation potential, the surface markers of MSCs are different. Compared to BMMSCs and AMSCs, human umbilical cord MSCs (HucMSCs) have higher levels of CD10, CD49d, CD54, CD200, and PDL2 expression, but lower levels of CD119, IFNγR1, and CD183 expression (29, 30). Coagulation factor III or tissue factor (TF)/CD142 levels were increased in AMSCs and PTMSCs compared to the BMMSCs (31, 32). Furthermore, the immunomodulatory properties of MSCs also differ. *In vitro* experiments showed that HucMSCs had a stronger inhibitory effect on T cell proliferation than placenta MSCs, followed by AMSCs and BMMSCs (33). However, according to other studies, BMMSCs exhibit more significant T cell suppressive capacity with increased expression of PDL1, IL10, and TGFβ1 (27, 34). Compared to BMMSCs and AMSCs, PTMSCs had the lowest expression of HLA antigens (HLA-DMA, HLA-DPB1, and HLA-DR) and immune-related genes (JAG1, TLR4, TLR3, NOTCH2, and NOTCH3), as well as reduced amounts of IL1α, IL6, and IL8 in the secretory group and increased IDO, IL1β, LIF and TNFβ2 (33, 35).

Studies have reported that MSCs act through their paracrine function and that MSCs are able to secrete a large number of RNAs, lipids, as well as a variety of soluble factors packaged in extracellular vesicles.

## 3 Biological characteristics of exosomes

Exosomes are a kind of extracellular vesicles with lipid bilayer membranes, which are cup-shaped structures under the microscope, ranging from 30-150 nm in diameter. Exosomes are formed in a process that involves double invagination of the plasma membrane and the formation of multivesicular bodies (MVBs). The plasma membrane is invaginated into a cup-like structure, thus forming early-sorting endosomes, which further mature into late-sorting endosomes with the involvement of the trans-Golgi network and endoplasmic reticulum (36–38). Meanwhile, intraluminal vesicles accumulate in late-sorting endosomes, converting them into MVBs. MVBs can be degraded by autophagosomes or lysosomes, or they can fuse with the plasma membrane to release intraluminal vesicles in the form of exosomes (29). Exosomes are taken up by recipient cells and participate in intercellular communication in three ways: receptor-ligand interaction, direct membrane fusion, and endocytosis/phagocytosis (39).

The nanoscale of exosomes allows them to be readily absorbed, cross the blood-brain barrier, and avoid being degraded by endosomes and lysosomal. While the lipid bilayer membrane structure can protect the cargo from degradation in the physical environment and provide long-term release effects to increase biological activity. Exosomes contain various molecular components, including DNA, RNA, lipids, and proteins derived from their parent cells, and are capable of performing important biological functions. In normal physiological processes, exosomes are able to participate in cell proliferation, immune regulation, neural communication, reproduction and development, and homeostasis. However, in pathological conditions, exosomes may interfere with immune responses, participate in the pathogenesis of tumors and neurodegenerative diseases, and even bring about pathogenic infections (39).

## 4 The roles of mesenchymal stem cell-derived exosomes in diabetes and its associated complications

### 4.1 Mesenchymal stem cell-derived exosomes and type 1 diabetes mellitus

T1DM is an autoimmune disease characterized by β-cells dysfunction and death, absolute insulin deficiency, and elevated blood glucose levels caused by autoreactive immune cells (40, 41). Therefore, inhibiting islet inflammation in T1DM and keeping a balance between auto-reactive effector T cells and regulatory T cells *in vivo* help to alleviate the injury and apoptosis of β-cells. Exosomes have been proven to have a robust immunomodulatory effect. Studies have reported that adipose-derived mesenchymal stem cell-derived exosomes (AMSC-Exos) exhibit immunomodulatory effects on T cells. The exosomes can also improve hyperglycemia symptoms in T1DM mice while increasing the number of regulatory T cells without affecting the proliferation index of lymphocytes (42). As the disease progresses, the number of pancreatic islets gradually decreases in T1DM patients due to inflammatory cells infiltrating the pancreatic islets and causing immune destruction of β-cells (43). Menstrual blood mesenchymal stem cell-derived exosomes (MenMSC-Exos) promote islet regeneration *via* the pancreas and duodenal homeobox one pathway, improve β-cells mass, and enhance insulin secretion in T1DM rats (44). Additionally, MSC-Exos have shown better therapeutic and regenerative effects than MSCs alone. Within four weeks of treatment with bone marrow mesenchymal stem cell-derived exosomes (BMMSC-Exos), T1DM rats showed a significant reduction in blood glucose levels and increased plasma insulin levels. Additionally, histopathology examination also revealed that the islets cells were regenerated, the number and size of Langerhans islets increased, while fibrosis and inflammation were reduced. However, the above indices were only slightly improved when the rats were treated with BMMSCs rather than BMMSC-Exos (45).

Islet transplantation is a prominent treatment for patients with T1DM. However, its application is limited by the shortage of organ donors and the loss of islets during the pre-transplant culture period and post-transplantation (46). Therefore, improving islet survival and function and preventing islet cell apoptosis are the key factors in islet transplantation. Vascular endothelial growth factor (VEGF), a pro-survival and anti-apoptotic factor that maintains islet mass, is expressed in islets but attenuated in isolated islets. Reduced expression of VEGF is associated with islet dysfunction and cell death. VEGF also facilitates the revascularization of transplanted islets, which is essential for long-term graft survival (47, 48). Exosomes from Wharton’s jelly mesenchymal stem cell (WJMSC-Exos) increase the expression of VEGF in co-cultured islets.

Furthermore, WJMSC-Exos can downregulate apoptotic genes such as BAD and BAX while upregulating the anti-apoptotic gene, BCL-2, and pro-survival gene, PI3K. PI3K can accelerate cell survival by activating Akt and inhibiting BAD and BAX (49), enhancing islet cell viability and reducing apoptosis. Proinflammatory cytokines trigger the expression of apoptosis and hypoxia-related genes, including iNOS, Fas, Caspase-3, and miR-375, causing immune rejection, and resulting in the destruction and dysfunction of transplanted islets. BMMSC-Exos can deliver siFAS and anti-miR-375, reduce immune activity and inhibit early apoptosis of transplanted islets (50). In the future, exosome therapy may eventually enable T1DM patients to reduce or eliminate insulin usage.

In addition to being utilized to treat T1DM, MSC-Exos may also be involved in its pathogenesis. According to *in vitro* research, islet MSC-like cells-derived exosomes (iMSC-Exos) are highly immunostimulatory. These exosomes may also contain specific antigens that stimulate autoreactive B cells, which in turn activate autoreactive T and B cells in prodromal diabetic NOD mice (a spontaneous disease model for T1DM) by binding to TLR. Additionally, the induction of auto-reactive Th1 cells was correlated with serum iMSC-Exos levels, with greater iMSC-Exos levels being linked to a rise in reactive Th1 cells. Therefore, iMSC-Exos may function as autoimmune triggers in NOD mice by acting as autoantigen carriers with strong adjuvant activity (51).

### 4.2 Mesenchymal stem cell-derived exosomes and type 2 diabetes mellitus and insulin resistance

T2DM, which accounts for 90% of patients with diabetes, is primarily caused by peripheral insulin resistance, pancreatic β-cells mass loss, and β-cells dysfunction (52). Glucose transporter 4 (GLUT4) is the major glucose transporter in adipose and skeletal muscle tissues and is strongly associated with peripheral insulin resistance in T2DM. GLUT4 translocation disorders and decreased expression reduce glucose uptake in skeletal muscle and adipose tissue, aggravating insulin resistance (53). In the T2DM rat model established by a high-fat diet and streptozotocin (STZ), human umbilical cord mesenchymal stem cell-derived exosomes (HucMSC-Exos) significantly ameliorated hyperglycemia in T2DM rats, restored insulin receptor substrate1 and the phosphorylation of protein kinase B (tyrosine site), and promoted the expression and membrane translocation of GLUT4 in skeletal muscle. Furthermore, HucMSC-Exos increased glycogen storage *in vivo* to maintain glucose homeostasis and inhibit STZ-induced β-cells apoptosis by restoring insulin secretion in T2DM rats (54). It was also reported that Langerhans islet induced atrophy and structural disorder in T2DM rats. A treatment using HucMSC-Exos restored islet structure, reduced the homeostatic model assessment of insulin resistance, and enhanced insulin sensitivity by promoting glucose uptake *via* GLUT1-4 in T2DM rats (55).

In conclusion, HucMSC-Exos can alleviate β-cells destruction and reverse peripheral insulin resistance. Hyperglycemia in diabetic patients encourages β-cells oxygen consumption, leading to β-cells hypoxia and a decline of PDX1 and MAFA, resulting in β-cells apoptosis and dysfunction (56, 57). HucMSC-Exos was shown to alleviate β-cells apoptosis under hypoxia by relieving hypoxia-induced endoplasmic reticulum stress and inhibiting p38 MAPK signaling in β-cells, which is mediated by the abundant miR-21 in HucMSC-Exos (58). Promoting islet regeneration and increasing insulin production is vital to restore T2DM. Umbilical cord blood mesenchymal stem cell-derived exosomes (UCBMSC-Exos) increased the proliferation of proliferative cells in Langerhans islets in STZ-induced diabetic mice, promoting pancreatic regeneration and improved insulin production by regulating the Extl3-Reg-cyclinD1 pathway (59). In summary, MSC-Exos can alleviate insulin resistance in insulin-sensitive tissues, such as the liver, muscle, and fat, and restore the function of β-cells.

### 4.3 Mesenchymal stem cell-derived exosomes and diabetic kidney disease

Diabetic kidney disease (DKD) is one of the severe microvascular complications of diabetes and a leading cause of end-stage renal disease worldwide (60). DKD pathogenesis involves various functional and structural changes, including hemodynamic changes, oxidative stress, mesangial cell expansion, and the development of glomerulosclerosis and fibrosis (61). The main clinical feature of DKD is persistent albuminuria, which can further develop into a decrease in glomerular filtration rate and renal tubular and interstitial lesions (62). Under diabetic conditions, almost all renal resident cells of DKD exhibit autophagy disorder (63). Exosomes from various tissues have been applied to treat DKD. BMMSC-Exos was reported to facilitate DKD by upregulating the autophagy function inhibited by the mTOR signaling pathway. Multiple injections of BMMSC-Exos significantly improved renal function in DKD mice, as observed in the significant decrease in serum creatinine (SCr), blood urea nitrogen (BUN), and urine albumin (UALB) levels. The injections also decreased the mesangial dilatation and improved renal fibrosis (64).

Exosomes contain abundant microRNAs (miRNAs), which play essential roles in immunoregulation, regulation of cell function, and homing mechanisms. miR-125a carried by AMSC-Exos mediated the protective effect of exosomes on DKD rats by inhibiting the HDAC1/ET1 axis (65). Meanwhile, miR-let-7a transported by BMMSC-Exos inhibited USP22 in the kidney tissue of DKD rats, leading to the decrease of SCr, BUN, triglycerides, and total cholesterol. Such action resulted in renal cells apoptosis and oxidative stress inhibition, and downregulation of N-cadherin and vimentin expression (66). In addition, BMMSC-Exos mediated its ameliorating effect on DKD rats by inhibiting the JAK2/STAT3 pathway (67).

Podocytes are terminally differentiated visceral epithelial cells that are an independent component of the glomerular filtration membrane, essential for maintaining glomerular filtration barrier function (68). In addition, miR-215-5p carried by AMSC-Exos attenuated high glucose-induced migration and injury in the mouse glomerular podocyte. It inhibited the expression of downstream ZEB2, alleviating podocyte injury and epithelial-mesenchymal transdifferentiation (69). BMMSC-Exos was also reported to inhibit STZ-induced apoptosis and degeneration of renal tubular epithelial cells in diabetic rats (70). Studies have shown that HucMSC-Exos carried abundant miR-146a-5p, which promotes M2 macrophage polarization by targeting the TRAF6-STAT1 signaling pathway to suppress renal inflammation and restore renal function (71). These findings suggest the possible role of miRNAs in mediating the amelioration of renal resident cells in the DKD model treated with various exosomes from various sources.

Urinary exosomes can be served as markers of DKD. In a study by Zubiri et al., it was found that the expression of regucalcin protein in renal tissue was reduced in diabetic kidneys, and this significant change can be reflected in urinary exosomes. Therefore, urinary exosomal regucalcin protein assay can be utilized for early diagnosis and progression monitoring of DKD (72). However, it is unclear whether urinary exosomes originate from urinary MSCs or from kidney-resident cells. For example, WT1 protein levels in urinary exosomes of patients with DKD increase with declining renal function, while WTI protein is also a marker and transcription factor for podocytes. Therefore, WT1 protein is likely to be derived from exosomes of podocytes rather than MSC-Exos (73).

### 4.4 Mesenchymal stem cell-derived exosomes and diabetic foot ulcer

Diabetic foot ulcer (DFU) is one of the leading causes of lower extremity amputation, which can be life-threatening in severe cases. About 6.3% of diabetic patients worldwide may develop diabetic foot diseases (74), with 17% and 5% requiring minor and major amputations, respectively, after one year (75). Diabetic neuropathy and peripheral vascular disease are the leading causes of DFU (76). The pathogenesis of DFU is multifactorial, with neuropathy, ischemia, and infection being the three main factors that are possibly combined with other factors such as diabetic skeletal disease, trauma, foot biomechanics, and weight bearing. Chronic wound healing in DFU is a complex dynamic physiological process that can be divided into four stages: hemostasis, inflammation, proliferation, and remodeling. Angiogenesis is critical in determining diabetic wound healing outcomes (77).

Multiple findings have indicated that BMMSC-Exos can enhance the proliferation and migration of fibroblasts, promoting diabetic wound healing (22, 78). Evangelos et al. found that BMMSC-Exos activated AKT, ERK 1/2, and STAT3 and induced the expression of many trophic factors (e.g., HGF, IL-6, IGF1, NGF, and SDF1) (22). In addition, Bi et al. reported that the therapeutic effect of exosomes on DFU models was mediated by the lncRNA H19 carried by BMMSC-Exos, which inhibited miR-152-3p and increased PTEN expression (78). Similarly, AMSC-Exos were found to improve the conditions of DFU models. It was reported that AMSC-Exos negatively regulated the expression of MMP1 and MMP3 in WS1, promoting collagen synthesis and wound healing. The treatment also inhibited cicatrix formation (79). Studies have also reported the role of lincRNA and miRNA in AMSCs-Exos in wound healing. A study demonstrated wound healing in diabetic mice promoted by induction of MIRI-128-3P/SIRT1-mediated autophagy with the overexpression of mmu_circ_0000250-modified AMSC-Exos (80). AMSC-Exos overexpressing linc00511 was also reported to accelerate angiogenesis and encourage healing of DFU through the inhibition of PAQR3-induced degradation of Twist1 ubiquitin (81). In one study, miR-21-5p mimics were loaded into AMSC-Exos by electroporation. The engineered AMSC-Exos promoted keratinocyte proliferation and migration *via* Wnt/β-catenin signaling *in vitro* and accelerated diabetic wound healing by increasing re-epithelialization, collagen remodeling, angiogenesis, and vascular maturation (82). Additionally, HucMSC-Exos can modulate endothelial cell function by reducing oxidative stress and inflammatory responses, promoting angiogenesis, and ultimately accelerating diabetic wound healing (83). Another study revealed that MenMSC-Exos facilitated wound healing primarily by promoting angiogenesis, enhancing re-epithelialization and wound closure rate, and altering the ratio of collagen type I/III (84). MSC-Exos participate in regulating the whole process of wound healing, treating diabetic foot ulcers. However, further research is required to determine the optimal way to incorporate exosomes in clinical applications.

### 4.5 The applications of mesenchymal stem cell-derived exosomes in other diabetic complications

MSC-Exos have also been applied in treating other diabetic complications, such as diabetic retinopathy, diabetic erectile dysfunction, diabetic osteoporosis, cognitive impairment, diabetic cardiomyopathy, and peripheral neuropathy, summarized in **Table 1**. The studies have demonstrated the role of MSC-Exos in ameliorating diabetic conditions and related complications.

**Table 1:** The role of mesenchymal stem cell-derived exosomes in other diabetic complications.

Diabetic complications	Exosomes sources	Mechanism of action	Effect	Ref
Diabetic retinopathy	HucMSCs	Alleviates retinal neuronal degeneration Inhibits microvascular disease Reduces the loss of neuronal cells and retinal vascular damage	Alleviates the disease progression	(85)
	HucMSCs	Activates the BDNF-TrkB pathway	Enhances neuronal cell viability and inhibit its apoptosis	(86)
	HucMSCs	Transports miR-17-3p and targets STAT1	Ameliorates retinal inflammation and oxidative damage	(87)
	BMMSCs	Down-regulates TLR4/NF-κB pathway and FBN1	Inhibits the oxidative stress, angiogenesis enhancement and inflammatory reaction Reduces retinal cell apoptosis	(88, 89)
	BMMSCs	Interacts with the miR-34a-5p/XBP1 signaling pathway	Inhibits human retinal microvascular endothelial cells endothelial-mesenchymal transition (EndMT) and capillaries angiogenesis	(90)
Erectile dysfunction	AMSCs	Restores the expression of cGMP by transporting corin enhances the expressions of nNOS, ANP and BNP	Improves the impaired neurovascular function Inhibits the expression of inflammatory factors	(91)
	AMSCs	Transfers pro-angiogenic microRNA and anti-fibrotic microRNA	Shows angiogenic properties and induces endothelial cell proliferation Reduces cavernous fibrosis and restores erectile function	(92)
	BMMSCs	Inhibits programmed cell death 4(PDCD4)	Promotes CCSMCs proliferation, and Inhibits CCSMCs apoptosis	(93)
Diabetic osteoporosis	BMMSCs	Transfers miR-140-3p Inhibits plexinB1/RhoA/ROCK signaling pathway	Promotes osteogenesis	(94)
	AMSCs	Suppresses the activation of NLRP3 inflammasome	Inhibits the production and secretion of pro-inflammatory cytokines and the bone resorption of osteoclasts	(95)
Cognitive impairment	BMMSCs	Suppresses oxidative stress and increased synaptic density Inhibits the proliferation of microglia in the brain Restores abnormal ultrastructure of neurons, astrocytes and blood vessels	Alleviates the disease progression	(96)
Diabetic cardiomyopathy	BMMSCs	Inhibits TGF-β1/Smad2 signaling pathway	Ameliorates DM-induced myocardial injury and fibrosis	(97)
Peripheral neuropathy	BMMSCs	Transfers miR-146a inhibits TLR4/NF-κB pathway	Increases the number of intraepidermal nerve fibers, myelin sheath thickness, and axonal diameter of the sciatic nerve Suppresses pro-inflammatory gene expression	(98, 99)

#### 4.5.1 Mesenchymal stem cell-derived exosomes and diabetic retinopathy

Diabetic retinopathy (DR) is a severe and long-term vision-impairing disease. DR is characterized by complications such as microaneurysms, exudates, hemorrhages, retinal neovascularization, and retinal edema. Because DR is considered a microvascular disease, various anti-VEGF approaches have been used clinically to prevent diabetic retinal neurovascular disease, including retinal neovascularization, vitreous hemorrhage, etc. However, anti-VEGF drugs cannot treat vision loss due to retinal ischemia and degeneration. Anti-VEGF drugs are also ineffective for treating early DR, although their effectiveness for advanced DR has been proven. A recent finding suggests that anti-VEGF may also impair neuronal survival and function (100).

Studies have shown that cell therapy using MSCs confers a specific protective effect on DR due to the immunomodulatory properties of MSCs (101, 102). However, due to ethical and safety issues related to MSCs transplantation, such as proliferative vitreoretinopathy, vitreous opacity, and vision loss (103), the treatment of DR with MSCs is still controversial. The therapeutic efficacy of MSCs is mainly attributable to paracrine-generated exosomes, whereby MSC-Exos have been shown to exert beneficial effects in ocular disease models (104–106). In recent years, researchers have reported that MSC-Exos could also significantly improve DR. Li et al. used real-time imaging methods such as fundus fluorescein angiography and optical coherence tomography to observe the fundus of rats. Combined with hematoxylin-eosin staining, it was observed that HucMSC-Exos could alleviate diabetes-induced retinal neuronal degeneration and inhibit microvascular disease. HucMSC-Exos also significantly reduced the loss of neuronal cells and retinal vascular damage in diabetic rats (107). A study revealed that HucMSC-Exos could transfer BDNF to retinal neurons and activate the BDNF-TrkB pathway to enhance high glucose (HG)-stimulated neuronal cell viability and inhibit its apoptosis (108). Another study reported that HucMSC-Exos transported miR-17-3p and ameliorated retinal inflammation and oxidative damage in DR mice by targeting STAT1 (109). Previous research has also revealed that BMMSC-Exos could inhibit oxidative stress, enhance angiogenesis, and induce an inflammatory reaction in the retinal cells of DR mice. The action is carried out by transporting miR-486-3p and miR-133b-3p through downregulation of the TLR4/NF-κB pathway and FBN1, respectively, resulting in inhibition of retinal cell apoptosis (110). In addition, Song et al. reported that BMMSC-Exos carrying the lncRNA SNHG7 could inhibit HG-stimulated human retinal microvascular endothelial cells endothelial-mesenchymal transition and capillaries angiogenesis by interacting with the miR-34a-5p/XBP1 signaling pathway (111).

#### 4.5.2 Mesenchymal stem cell-derived exosomes and erectile dysfunction

Erectile dysfunction is one of the major complications of diabetes, occurring in about 50% of men with diabetes within ten years of diagnosis (112). Various factors contribute to diabetic erectile dysfunction (DED), including hyperglycemia, hypertension, hyperlipidemia, insulin resistance, androgen deficiency, and vascular and neuronal abnormalities (113, 114). Mechanisms that mediate DED mainly include increased advanced glycation end products, impaired neuronal nitric oxide synthase (nNOS) synthesis, elevated levels of oxygen free radicals, and decreased levels of cyclic guanosine monophosphate (cGMP)-dependent kinase-1 and Nitric oxide (NO). Endothelial dysfunction is the primary pathophysiology of DED, which is characterized by the disability of the endothelium to generate vasodilatory messengers and maintain vasodilation and vascular homeostasis (115, 116). Growing evidence suggests that DED is a vascular disease (117).

AMSCs have attracted considerable attention as a practical cell transplantation resource for treating DED. Previous studies have demonstrated that the NO-cGMP pathway plays a vital role in maintaining normal erectile function since the loss of cGMP leads to DED (118). AMSC-Exos restored the expression of cGMP by transporting corin and enhanced the expressions of nNOS, ANP, and BNP. This action improved the impaired neurovascular function of DM rats and inhibited the expression of inflammatory factors, reversing the DED conditions caused by DM (119). Part of the pathophysiology of DED involves a contraction-relaxation imbalance of smooth muscle cells (SMCs). Cavernous SMCs (CCSMCs) have been reported to modulate a phenotypic transition from a contractile to a proliferative state under hyperglycemic conditions, which may play an essential role in the pathogenesis of DED (120). Li et al. reported that BMMSC-Exos could transport miR-21-5p to inhibit programmed cell death 4 (PDCD4), promote CCSMCs proliferation, and inhibit CCSMCs apoptosis, improving DED in the DM rat model (121).

#### 4.5.3 Mesenchymal stem cell-derived exosomes and diabetic osteoporosis

Growing evidence suggests a strong interaction between glucose levels and changes in bone metabolism. Impaired bone healing is considered a significant complication associated with DM. T2DM also causes changes in bone metabolism, impairs bone quality, and results in decreased bone strength, increased fracture risk, and impaired bone healing (122, 123). It was reported that the duration of fracture healing in DM patients is prolonged by 87% (124). BMMSCs are considered to be a promising source of engineered tissue cells that not only exhibit osteogenic differentiation properties but can also stimulate osteogenesis required for bone regeneration (125). Several studies have shown that exosomes mediate the role of MSCs in promoting osteogenesis and potentially regulating bone metabolism (126, 127). Furthermore, MSCs-Exos are more stable than MSCs for therapeutic intervention in specific physiological environments (128).

Studies have revealed that BMMSC-Exos also mediate the effect of BMMSCs in promoting osteogenesis, offering excellent potential in regulating bone metabolism. Wang et al. reported that both BMMSC-Exos of normal rats and BMMSC-Exos of diabetic rats could promote osteoblastogenesis and mineralization in BMMSCs of normal and DM rats, respectively. Additionally, Normal-BMMSC-Exos have demonstrated a more significant osteogenic effect on osteoblastogenesis than DM-BMMSC-Exos. Further studies found that BMMSC-Exos transfer miR-140-3p and miR-140-3p and promote osteogenesis by inhibiting the plexinB1/RhoA/ROCK signaling pathway (129). Inflammation is one of the leading causes of diabetic osteoporosis. Cheng et al. found that AMSC-Exos could inhibit the production and secretion of proinflammatory cytokines (e.g., IL-18 and IL-1β) in osteoclasts and inhibit the bone resorption by osteoclasts through the inactivation of NLRP3 inflammasome (130, 131). Studies have also revealed that AMSC-Exos overexpressing miR-146a exhibits a more substantial inhibitory effect than normal AMSC-Exo, whereas miR-146a-Exo inhibits the activation of the inflammasome by inhibiting HG-related osteoclast inflammatory response, improving diabetic osteoporosis *in vivo*.

#### 4.5.4 Mesenchymal stem cell-derived exosomes and cognitive impairment

Diabetes-related cognitive impairment is a global challenge, with epidemiological studies reporting that the incidence of dementia in diabetic patients is two to three times higher than in nondiabetic patients. Multiple mechanisms are thought to contribute to diabetes-related dementia, including abnormal glucose metabolism (e.g., hyperglycemia and hypoglycemia), abnormal insulin action (e.g., insulin deficiency and insulin resistance), vascular abnormalities, and oxidative stress in the central nervous system (85, 132). The damage to hippocampal neurons and astrocytes is considered the most critical problem of cognitive impairment caused by diabetes. Mineko Fujimiya et al. reported that BMMSC-Exos could improve learning and memory impairment in diabetic mice. It was also reported that BMMSC-Exos do not increase the number of neurons but suppress oxidative stress and increase synaptic density while inhibiting the proliferation of microglia in the brain from restoring the abnormal ultrastructure of neurons, astrocytes, and blood vessels (86).

#### 4.5.5 Mesenchymal stem cell-derived exosomes and diabetic cardiomyopathy

Cardiovascular disease is the leading cause of death in diabetic patients, including diabetic cardiomyopathy. The main characteristics of diabetic cardiomyopathy include myocardial fibrosis, chronic inflammation, and cardiac structure and function changes caused by chronic hyperglycemia in the absence of myocardial ischemia and hypertensive heart disease (87, 88). Lin et al. demonstrated that BMMSC-Exos could ameliorate DM-induced myocardial injury and fibrosis by inhibiting the TGF-β1/Smad2 signaling pathway (89).

#### 4.5.6 Mesenchymal stem cell-derived exosomes and peripheral neuropathy

Diabetic peripheral neuropathy (DPN) is one of the common chronic complications of diabetes (90). By 2030, around 50 million people worldwide will suffer from DPN (91). Therefore, there is an urgent need to develop effective therapies designed to improve DPN. Liu et al. reported that BMMSC-Exos significantly reduced thermal and mechanical stimulation thresholds and increased nerve conduction velocity in DPN mice. Histopathological analysis revealed that BMMSC-Exos significantly increased the density of FITC-dextran-perfused vessels and increased the number of intraepidermal nerve fibers, myelin sheath thickness, and axonal diameter of the sciatic nerve. BMMSC-Exos could also attenuate neurovascular dysfunction and facilitate functional recovery in DPN mice by inhibiting TLR4/NF-κB pathway and suppressing proinflammatory gene expression. Notably, studies have shown that BMMSC-Exos overexpressing miR-146a amplifies the therapeutic effect in DPN compared to normal exosomes (92, 93).

## 5 Mesenchymal stem cell-derived exosomes for drug delivery

The therapeutic roles of MSC-Exos in diabetes and related complications were summarized above. More and more studies demonstrated that MSC-Exos can be applied as carriers for drugs in addition to miRNAs, proteins, and other cargoes. The lipid bilayer membrane structure of MSC-Exos can protect the integrity and the biological activity of drugs, and the membrane modification of MSC-Exos can achieve targeted drug delivery. Additionally, the low immunogenicity, low tumorigenicity, and high biocompatibility of MSC-Exos guarantee the safety of treatment. Exosomes have been explored to load different drugs to treat various diseases. Other researchers load chemotherapy drugs such as adriamycin and paclitaxel into MSC-Exos for the treatment of tumor diseases with reduced drug side effects (133, 134). In diabetes treatment, miR-21-5p mimics were loaded into AMSC-Exos for diabetic wound healing (82). In the future, it is intended to investigate MSC-Exos’ potential to carry more potent drugs for the treatment of diabetes and related complications.

## 6 Which component in exosomes mediates the therapeutic effects?

**Figure 1:**
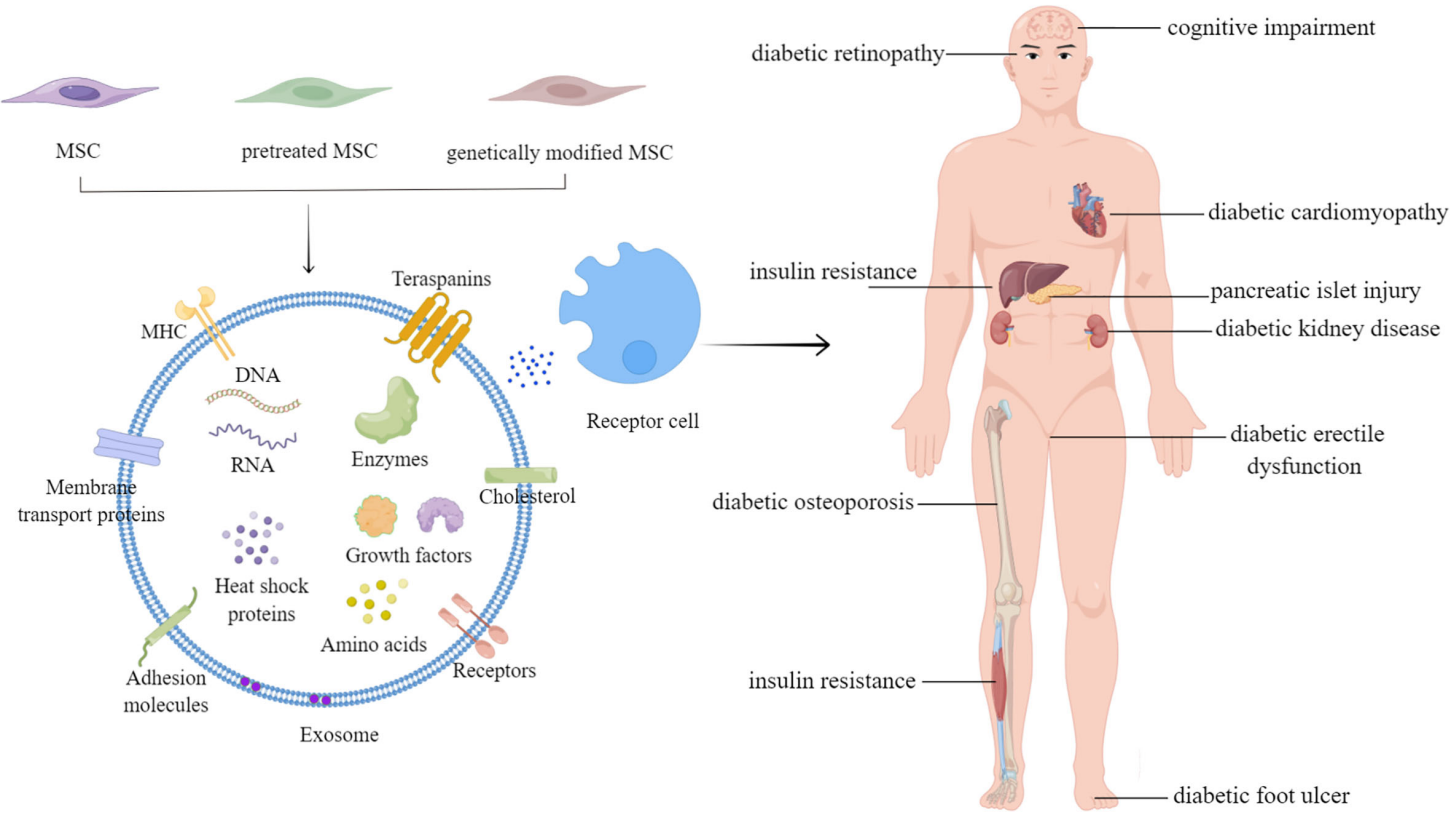
The applications of mesenchymal stem cell-derived exosomes in diabetes mellitus and related complications.

Various cellular components, including RNAs (e.g., mRNAs, miRNAs, lincRNAs, etc.), DNAs, proteins, and lipids, are contained in exosomes (135) (**Figure 1**, left panel). However, nearly all current research has suggested that proteins and RNAs mediate the process of exosomes, whereas DNAs and lipids have received relatively little attention. MiRNAs are particularly prevalent in the research on several types of exosomal RNAs. Many studies have performed deep miRNA sequencing on MSC-Exos from various sources, or MSC-Exos that have been pretreated and genetically modified, to explore the roles of different miRNAs in different MSC-Exos for the treatment of other diseases (100, 101, 136). MiRNAs transported by exosomes serve critical roles in modulating the processes for treating diabetes and related complications. However, according to Toh et al., the concentration of pre-miRNAs in exosomes is inadequate to trigger biological reactions. Additionally, miRNAs are only biologically active when they bind to RNA-induced silencing complexes, yet exosomes typically lack the full RNA-induced silencing complexes.

Meanwhile, biological processes can be triggered by the concentration of proteins in exosomes at therapeutic doses, exhibiting ATP generation by a glycolytic enzyme, which suggests that proteins might be the primary driver of MSC-Exos therapeutic activity (102). With the development of proteomics technology, scientists can now identify the composition of proteins in exosomes and further analyze their functions. Numerous studies have identified the proteins in MSC-Exos from various sources. The proteomics of BMMSC-Exos, AMSC-Exos, and HucMSC-Exos was systematically and comprehensively researched, leading to the discovery of 431, 457, and 771 proteins, respectively. Bioinformatics analysis of the shared and unique proteins of the three exosomes revealed that HucMSC-Exos have remarkable tissue damage healing capacity.

Additionally, the study suggested the regeneration ability of BMMSC-Exos and the role of AMSC-Exos in immune regulation (103). Proteomics has been used to research the functions of exosomes in various disorders, including heart attacks, pulmonary fibrosis, and Alzheimer’s disease (104–106). Despite the application of proteomics, it is important to determine which protein is crucial in the pathogenesis of a disease.

In conclusion, the prevailing theory holds that exosomes play a critical role in controlling intercellular communication *via* proteins and miRNAs. There is a need for a comprehensive investigation of the mechanism of exosomes in treating diabetes and its related complications. Similarly, the roles of DNAs and lipids in exosomes cannot be disregarded.

## 7 How do we improve the therapeutic effects of exosomes?

As mentioned earlier, exosomes from various tissues have various therapeutic effects on diabetes and related complications. However, the paracrine action of MSCs may be easily affected in a physicochemical environment due to the potential replicative senescence and reduced stability during the passage process, which reduces their therapeutic efficiency. Therefore, it is vital to identify the correct approach to enhance the therapeutic effect of exosomes. Researchers have found that MSC-Exos could deliver a superior therapeutic effect when subjected to specific pretreatments or genetic modifications. In addition, biomaterials can be used to encapsulate exosomes to strengthen their controlled-release effect and broaden their therapeutic effects (**Table 2**).

**Table 2:** The different exosome engineering strategies.

Diabetic complications	Exosomes sources	Pretreatment/Genetic modification/Biomaterials	Ref
Diabetic foot ulcer	AMSCs	Overexpressed mmu_circ_0000250	(80)
	AMSCs	Overexpressed linc00511	(81)
	AMSCs	Loaded miR-21-5p mimics	(82)
	AMSCs	Hypoxia pretreated	(110)
	BMMSCs	Atorvastatin pretreated	(114)
	BMMSCs	Pioglitazone pretreated	(115)
	BMMSCs	Melatonin pretreated	(118)
	HucMSCs	Lipopolysaccharide pretreated	(119)
	HucMSCs	Nanohydrogel	(122)
	AMSCs	Hydrogel	(120)
	Synovium MSCs	Chitosan/overexpressed miR-126-3p	(124)
	Gingival mesenchymal stem cells	Chitosan/silk hydrogel	(125)
Diabetic osteoporosis	AMSCs	Overexpressed miR-146a	(137)
Peripheral neuropathy	BMMSCs	Overexpressed miR-146a	(99)

Previous studies have shown that the pretreatment of MSCs with physical, chemical, and biological factors effectively enhances the biological activity of MSC-Exos and improves their repair efficacy in tissue engineering and regenerative medicine (107). For example, exosomes extracted from IL-1β-pretreated MSCs exhibited a higher IL-10 and TGF-β than exosomes extracted from untreated MSCs (108). In addition, hypoxia pretreatment increases angiogenesis and neuroprotection, improves MSCs proliferation and migration, and enhances MSCs engraftment efficiency compared to MSCs under normal culture conditions (109). Compared with normoxic exosomes, 215 miRNAs were upregulated, and 369 were downregulated in hypoxic AMSC-Exos. The upregulated miR-21-3p/miR-126-5p/miR-31-5p and the downregulated miR-99b/miR-146-a could activate the relevant signaling pathways, promoting fibroblast proliferation and migration (110). The activation of the PI3K/Akt/eNOS pathway is a key process in stimulating angiogenesis, enhancing human umbilical vein endothelial cells viability, and promoting capillary formation (111). NO produced by eNOS is a vasoactive substance secreted by the vascular endothelial system, which plays a vital role in maintaining vascular homeostasis (112). The activation of PI3K/Akt leads to phosphorylation of eNOS, resulting in NO production. Conversely, inhibition of the PI3K/Akt pathway results in reduced eNOS phosphorylation and inhibition of NO production, which is associated with endothelial cell dysfunction (113). Additionally, exosomes of BMMSCs pretreated with atorvastatin and pioglitazone can activate the PI3K/AKT/eNOS pathway, enhance the biological function of human umbilical vein endothelial cells, and promote neovascularization (114, 115). Macrophage polarization plays an important role in diabetic wound healing. M1 macrophages produce proinflammatory cytokines such as IL-1β and TNF-α, which may lead to organ dysfunction. Meanwhile, M2 macrophages are related to the production and secretion of anti-inflammatory cytokines, associated with reduced inflammatory response (116). An increase in the M2 phenotype and a decrease in the M1 phenotype contribute to diabetic wound repair (117). In addition, both melatonin-pretreated BMMSC-Exos and lipopolysaccharide-pretreated HucMSC-Exos can enhance the polarization of macrophages toward the M2 phenotype, thereby inhibiting the inflammatory response and promoting diabetic wound healing (118, 119).

Exosomes are typically injected to treat diabetic foot wound healing, but infection and other factors could aggravate even a tiny wound. However, achieving good treatment outcomes is challenging because exosomes are quickly cleared and easily inactivated *in vitro*. Therefore, researchers have designed various biomaterials to encapsulate and protect exosomes, improving their effectiveness and maintaining the moisture level in damaged tissues (120, 121).

Mei et al. demonstrated that the exosomes encapsulated in hydrogel could effectively promote skin cell proliferation, migration, and angiogenesis and accelerate the healing of diabetic wounds compared with the non-encapsulated exosome (120, 122, 123). A novel nanohydrogel (NH) composed of polyvinyl alcohol (PVA) and alginate (Alg) was combined with HucMSC-Exos to form an Exo@H complex. This complex could activate the ERK1/2 signaling pathway and elevate SMA, CD31, SR-B1, and VEGF expression in diabetic wound, mediating diabetic wound healing (122). An injectable, self-healing, and antibacterial polypeptide-based FHE hydrogel encapsulating AMSC-Exos has been demonstrated to accelerate granulation tissue formation, re-epithelialization, and collagen remodeling at the wound site. Compared with the FHE hydrogel group, the pure exosomes group, and the control group, the skin appendages were more abundant, and the scar tissue was reduced in the FHE@exo hydrogel group (120). Zhang et al. used chitosan to encapsulate exosomes derived from synovial mesenchymal stem cells overexpressing miR-126-3p (SMSC-126-Exos). They found that SMSC-126-Exos could significantly activate PI3K/AKT and MAPK/ERK pathways compared with pure SMSC-Exos.

Moreover, the controlled release of SMSC-126-Exos significantly increased re-epithelialization and collagen deposition at the wound site, activating and promoting neovascular maturation (124). In addition, a chitosan/silk hydrogel sponge biomaterial has also been used as a support for exosomes derived from gingival MSCs. The exosome-hydrogel complexes could promote the re-epithelialization, deposition, and remodeling of the extracellular matrix. These complexes also promote angiogenesis and neuronal ingrowth, accelerating cutaneous wound healing in STZ-induced diabetic rats (125).

Unfortunately, there are no universal pretreatments of MSCs that can be beneficial for the therapy of different diabetic complications. Researchers should further investigate the mechanisms of exosomes in ameliorating diseases and explore the potential alternatives to modify the current methods.

## 8 Challenges and prospects

Diabetes and its complications can be treated effectively using MSC-Exos, as described in the preceding sections. However, it is necessary to resolve several issues before incorporating MSC-Exos in treating diabetes.

One key challenge in treating diabetes with MSC-Exos is the effect of residing tissues on MSC-Exos. According to previous studies, when exosomes were infused through the tail vein, only a few remained in the liver and spleen for 24 or 48 h (126, 127), probably because the liver and spleen are rich in blood. Therefore, it is crucial to explore the mechanisms of how MSC-Exos homing targets tissues and how it plays a long-term therapeutic role. It is interesting to know whether damaged target tissues secrete chemokines and cytokines that guide exosome homing *in vivo*. Several studies have revealed that MSCs can release various mediators, including immunosuppressive molecules, growth factors, exosomes, chemokines, complement components, and multiple metabolites, to regulate the inflammatory balance and microenvironment of damaged tissues when exposed to inflammatory environments (128). Exosomes may be dynamic depending on the state of the residing tissue, such as immunomodulation, tissue damage repair, or regeneration. It would be ideal for MSC-Exos to encourage angiogenesis when treating DFU but block it when treating DR. The finding suggests investigating why the same type of MSC-Exos can have opposing effects in various injured tissues.

Additionally, the controlled-release MSC-Exos technology applications in diabetes only apply to diabetic foot ulcers. There is a need to optimize the targeting and sustained-release technology of MSC-Exos *in vivo* to treat diabetic complications like the pancreatic injury, DKD and others. This optimization will prevent MSC-Exos from being destroyed by the physicochemical environment *in vivo* and help them play a long-term effect.

Secondly, MSC-Exos have been showing significant heterogeneity. MSC-Exos derived from different tissues have unique characteristics. Exosomes from different tissues have different proteomic characteristics, which can be used as a guide when choosing which exosomes to use for disease treatment (103, 129). Furthermore, exosomes from various tissue sources may have different therapeutic effects on the same disease. The use of MSCs from the same tissue source can rarely ensure the standardization of MSC-Exos content and their therapeutic effects on diseases because of the varied culture conditions, such as medium, cell production, and dimensions of the culture environment (two-dimensional or three-dimensional). Additionally, different exosome extraction methods can also cause heterogeneity in its contents. Therefore, when carrying out MSC-Exos therapy, it is important to consider an approach that can maximize the effectiveness of exosome therapy, i.e., ensuring the consistency in exosome isolation methods and the sources of exosomes, etc.

Finally, further clinical investigations are required to verify the therapeutic effect of MSC-Exos on diabetes and its associated complications. There have already been some clinical trials using MSC-Exos to treat these diseases (available online: http://www.clinicaltrials.gov/) (**Table 3**). In one of the clinical trials using UCBMSC-EV in chronic kidney disease, it was demonstrated that administration of UCBMSC-EV was safe and the treatment improved the inflammatory immune response and overall renal function in patients with grade III-IV CKD, as evidenced by significant improvements in eGFR, Scr, Bun and UACR, increases in plasma TGF-β1 and IL-10, and decreases in TNF-α plasma levels in these patients (130). Therefore, it is critical to conduct additional fundamental research and clinical trials. These studies will result in the discovery of novel treatments for diabetes and its related complications.

**Table 3:** Clinical trial of MSC-Exos in the treatment of diabetes and related complications.

Disease	Intervention	Follow up	Location	State	ClinicalTrials.gov Identifier
Diabetes Mellitus Type 1	UCBMSC- microvesicles	3 months	Sahel Teaching Hospital Sahel, Cairo, Egypt, 11522	Unkown	NCT02138331
Foot, Diabetic	Personalized Nutritional Intervention and MSC-Exos	1/2/3 months	Hospital Universitario Reina Sofía de Córdoba Córdoba, Andalucía, Spain, 14004	Not yet recruiting	NCT05243368
Macular Holes	MSC-Exos	24 weeks	Tianjin Medical University Hospital Tianjin, China	Active, not recruiting	NCT03437759
Chronic Kidney Disease	UCBMSC- extracellular vesicles	1 year	Sahel Teaching Hospital Sahel, Cairo, Egypt, 11522	Concluded	(130)

In addition to treating diseases, exosomes can be employed as natural drug carriers to deliver drugs, such as siRNAs, shRNAs, and other molecules. Modification of exosomes can enhance the therapeutic effect and targeting ability. For example, miR-21-5p mimics were electroporated into AMSC-Exos, exploiting the natural availability and biocompatibility of exosomes as extracellular miRNA transport particles to promote diabetic cutaneous wound healing (82). Exosomes carrying diverse therapeutic drugs may perform crucial therapeutic functions in the future.

Numerous nanovesicles can be generated by cell extrusion or polymer nanoparticles coated with cell membranes. These nanovesicles are called exosome mimetic vesicles (EMVs). While exosomes produced by cells are limited, EMVs are abundant. EMVs are comparable to exosomes in size, structure, and components and can also participate in cell-to-cell communication (131). Proteomic analysis of EMVs and exosomes derived from HucMSCs revealed that they share about 80% of the same protein while also containing unique proteins. EMVs and exosomes derived from HucMSCs have tissue repair ability, promoting wound healing and angiogenesis in mice (132). Future research must determine whether different MSC-derived EMVs have the same therapeutic effect as exosomes and whether they can be applied to diabetes and associated complications and other diseases.

## Conclusion

Diabetes is a chronic condition that severely threatens global public health. This review outlines the roles and mechanisms of MSC-Exos in treating diabetes and its related complications (**Figure 1**). MSC-Exos have shown promising results in treating diabetes and may develop into a successful strategy for treating diabetes and complications in the future.
