# Correction: The roles of mesenchymal stem cell-derived exosomes in diabetes mellitus and its related complications

**DOI:** 10.3389/fendo.2026.1852895

**Published:** 2026-08-03

**Authors:** Mengmeng Yang, Jun Chen, Li Chen

**Affiliations:** 1 Department of Endocrinology, Qilu Hospital, Shandong University, Jinan, China; 2 Institute of Endocrine and Metabolic Diseases of Shandong University, Jinan, China; 3 Key Laboratory of Endocrine and Metabolic Diseases, Shandong Province Medicine & Health, Jinan, China; 4 Jinan Clinical Research Center for Endocrine and Metabolic Diseases, Jinan, China

**Keywords:** diabetes mellitus, complications, mesenchymal stem cells, exosomes, β-cell

In the original version of the article, four references have been withdrawn, and we want to remove the withdrawn references which were numbered 69, 112, 122 and 136.

There was a mistake in **Table 2** as published. Two references in **Table 2** have been withdrawn. The corrected **Table 2** appears below.

**Table 2:** The different exosome engineering strategies.

Diabetic complications	Exosomes sources	Pretreatment/Genetic modification/Biomaterials	Ref
Diabetic foot ulcer	AMSCs	Overexpressed mmu_circ_0000250	(81)
	AMSCs	Overexpressed linc00511	(82)
	AMSCs	Loaded miR-21-5p mimics	(83)
	AMSCs	Hypoxia pretreated	(111)
	BMMSCs	Atorvastatin pretreated	(116)
	BMMSCs	Pioglitazone pretreated	(117)
	BMMSCs	Melatonin pretreated	(120)
	HucMSCs	Lipopolysaccharide pretreated	(121)
	HucMSCs	Nanohydrogel	(125)
	AMSCs	Hydrogel	(123)
	Synovium MSCs	Chitosan/overexpressed miR-126-3p	(127)
	Gingival mesenchymal stem cells	Chitosan/silk hydrogel	(128)
Diabetic osteoporosis	AMSCs	Overexpressed miR-146a	(141)
Peripheral neuropathy	BMMSCs	Overexpressed miR-146a	(100)

Due to the removal of four withdrawn references, the contents of the references corresponding to the articles also need to be removed.

Correction have been made to the following sections:

Section 4 “**The roles of mesenchymal stem cell-derived exosomes in diabetes and its associated complications**”, subsection 4.3 “*Mesenchymal stem cell-derived exosomes and diabetic kidney disease*”, Paragraph 3:

“Podocytes are terminally differentiated visceral epithelial cells that are an independent component of the glomerular filtration membrane, essential for maintaining glomerular filtration barrier function (68). In addition, miR-215-5p carried by AMSC-Exos attenuated high glucose-induced migration and injury in the mouse glomerular podocyte. It inhibited the expression of downstream ZEB2, alleviating podocyte injury and epithelial-mesenchymal transdifferentiation (70). BMMSC-Exos was also reported to inhibit STZ-induced apoptosis and degeneration of renal tubular epithelial cells in diabetic rats (71). Studies have shown that HucMSC-Exos carried abundant miR-146a-5p, which promotes M2 macrophage polarization by targeting the TRAF6-STAT1 signaling pathway to suppress renal inflammation and restore renal function (72). These findings suggest the possible role of miRNAs in mediating the amelioration of renal resident cells in the DKD model treated with various exosomes from various sources.”

Section 7 **How do we improve the therapeutic effects of exosomes?** Paragraph 2:

“Previous studies have shown that the pretreatment of MSCs with physical, chemical, and biological factors effectively enhances the biological activity of MSC-Exos and improves their repair efficacy in tissue engineering and regenerative medicine (108). For example, exosomes extracted from IL-1β-pretreated MSCs exhibited a higher IL-10 and TGF-β than exosomes extracted from untreated MSCs (109). In addition, hypoxia pretreatment increases angiogenesis and neuroprotection, improves MSCs proliferation and migration, and enhances MSCs engraftment efficiency compared to MSCs under normal culture conditions (110). Compared with normoxic exosomes, 215 miRNAs were upregulated, and 369 were downregulated in hypoxic AMSC-Exos. The upregulated miR-21-3p/miR-126-5p/miR-31-5p and the downregulated miR-99b/miR-146-a could activate the relevant signaling pathways, promoting fibroblast proliferation and migration (111). The activation of the PI3K/Akt/eNOS pathway is a key process in stimulating angiogenesis, enhancing human umbilical vein endothelial cells viability, and promoting capillary formation (113). NO produced by eNOS is a vasoactive substance secreted by the vascular endothelial system, which plays a vital role in maintaining vascular homeostasis (114). The activation of PI3K/Akt leads to phosphorylation of eNOS, resulting in NO production. Conversely, inhibition of the PI3K/Akt pathway results in reduced eNOS phosphorylation and inhibition of NO production, which is associated with endothelial cell dysfunction (115). Additionally, exosomes of BMMSCs pretreated with atorvastatin and pioglitazone can activate the PI3K/AKT/eNOS pathway, enhance the biological function of human umbilical vein endothelial cells, and promote neovascularization (116, 117). Macrophage polarization plays an important role in diabetic wound healing. M1 macrophages produce proinflammatory cytokines such as IL-1β and TNF-α, which may lead to organ dysfunction. Meanwhile, M2 macrophages are related to the production and secretion of anti-inflammatory cytokines, associated with reduced inflammatory response (118). An increase in the M2 phenotype and a decrease in the M1 phenotype contribute to diabetic wound repair (119). In addition, both melatonin-pretreated BMMSC-Exos and lipopolysaccharide-pretreated HucMSC-Exos can enhance the polarization of macrophages toward the M2 phenotype, thereby inhibiting the inflammatory response and promoting diabetic wound healing (120, 121).”

Section 4 **The roles of mesenchymal stem cell-derived exosomes in diabetes and its associated complications,** subsection 4.5 “*The applications of mesenchymal stem cell-derived exosomes in other diabetic complications,*” subsection 4.5.2 “*Mesenchymal stem cell-derived exosomes and erectile dysfunction,*” Paragraph 2:

“AMSCs have attracted considerable attention as a practical cell transplantation resource for treating DED. Previous studies have demonstrated that the NO-cGMP pathway plays a vital role in maintaining normal erectile function since the loss of cGMP leads to DED (120). AMSC-Exos restored the expression of cGMP by transporting corin and enhanced the expressions of nNOS, ANP, and BNP. This action improved the impaired neurovascular function of DM rats and inhibited the expression of inflammatory factors, reversing the DED conditions caused by DM (121). Part of the pathophysiology of DED involves a contraction-relaxation imbalance of smooth muscle cells (SMCs). Cavernous SMCs (CCSMCs) have been reported to modulate a phenotypic transition from a contractile to a proliferative state under hyperglycemic conditions, which may play an essential role in the pathogenesis of DED (123). Li et al. reported that BMMSC-Exos could transport miR-21-5p to inhibit programmed cell death 4 (PDCD4), promote CCSMCs proliferation, and inhibit CCSMCs apoptosis, improving DED in the DM rat model (124).”

Section 5 **Mesenchymal stem cell-derived exosomes for drug delivery,** Paragraph 1:

“The therapeutic roles of MSC-Exos in diabetes and related complications were summarized above. More and more studies demonstrated that MSC-Exos can be applied as carriers for drugs in addition to miRNAs, proteins, and other cargoes. The lipid bilayer membrane structure of MSC-Exos can protect the integrity and the biological activity of drugs, and the membrane modification of MSC-Exos can achieve targeted drug delivery. Additionally, the low immunogenicity, low tumorigenicity, and high biocompatibility of MSC-Exos guarantee the safety of treatment. Exosomes have been explored to load different drugs to treat various diseases. Other researchers load chemotherapy drugs such as adriamycin and paclitaxel into MSC-Exos for the treatment of tumor diseases with reduced drug side effects (137, 138). In diabetes treatment, miR-21-5p mimics were loaded into AMSC-Exos for diabetic wound healing (83). In the future, it is intended to investigate MSC-Exos’ potential to carry more potent drugs for the treatment of diabetes and related complications.”

The original version of this article has been updated.

